# Neotropical peatland methane emissions along a vegetation and biogeochemical gradient

**DOI:** 10.1371/journal.pone.0187019

**Published:** 2017-10-20

**Authors:** R. Scott Winton, Neal Flanagan, Curtis J. Richardson

**Affiliations:** Duke University Wetland Center, Nicholas School of the Environment, Duke University, Durham, NC, United States of America; University of Copenhagen, DENMARK

## Abstract

Tropical wetlands are thought to be the most important source of interannual variability in atmospheric methane (CH_4_) concentrations, yet sparse data prevents them from being incorporated into Earth system models. This problem is particularly pronounced in the neotropics where bottom-up models based on water table depth are incongruent with top-down inversion models suggesting unaccounted sinks or sources of CH_4_. The newly documented vast areas of peatlands in the Amazon basin may account for an important unrecognized CH_4_ source, but the hydrologic and biogeochemical controls of CH_4_ dynamics from these systems remain poorly understood. We studied three zones of a peatland in Madre de Dios, Peru, to test whether CH_4_ emissions and pore water concentrations varied with vegetation community, soil chemistry and proximity to groundwater sources. We found that the open-canopy herbaceous zone emitted roughly one-third as much CH_4_ as the *Mauritia flexuosa* palm-dominated areas (4.7 ± 0.9 and 14.0 ± 2.4 mg CH_4_ m^-2^ h^-1^, respectively). Emissions decreased with distance from groundwater discharge across the three sampling sites, and tracked changes in soil carbon chemistry, especially increased soil phenolics. Based on all available data, we calculate that neotropical peatlands contribute emissions of 43 ± 11.9 Tg CH_4_ y^-1^, however this estimate is subject to geographic bias and will need revision once additional studies are published.

## Introduction

Inverse modelling reveals that tropical wetlands account for much of the variability in global atmospheric concentrations of the important greenhouse gas, methane (CH_4_) [1]. Yet tropical wetlands remain absent from Earth system models because of a scarcity of ground-based data to parameterize bottom-up models, making these ecosystems a ‘missing link’ in the global carbon cycle [2]. In the Amazon basin, for example, modelled bottom-up source strength based on water table depth cannot explain top-down detected emissions patterns [3]. Some researchers speculate that cryptic wetlands [4] or vegetation sources [5] may account for this discrepancy, but the recent finding that perennially flooded peatlands in South America cover three to four times more area than previously realized [6] may point to a missing and/or misunderstood Amazon CH_4_ source.

Although CH_4_ emissions from high latitude peatlands have been well-studied [7], relatively little data have been published from the tropics and almost none exists for South America [8], yet this continent is now thought to contain the largest area of tropical peatland cover [6]. The scarcity of ground-based CH_4_ data from neotropical peatlands is the major reason why these important ecosystems are not included in many regional or global scale greenhouse gas models [2].

Studies of wetland CH_4_ dynamics from other regions have revealed generalizable patterns that provide a framework for how Amazonian peatlands might function. First, emissions differences within and between wetlands tend to be governed primarily by hydrology, which determines soil redox conditions [9]. Second, plant biomass and carbon quality directly related to the amount of available substrate for methanogenic microorganisms, and thus CH_4_ emissions often varies across vegetation zones [10]. Third, it has been shown that peat containing higher concentrations of plant-derived phenolics will block peat decomposition and greenhouse gas emissions [11,12]. And finally, ombrotropic peatlands tend to emit less CH_4_ than minerotrophic wetlands because of inhibitory effects of low pH [13]. It is worth retesting these assumptions in the context of Amazonian peatlands because of their potential importance to global CH_4_ budgets and because they are so poorly studied.

In this paper we measured CH_4_ emissions and soil chemistry from the most pervasive peatland community type in Amazonia, the *Mauritia flexuosa* palm swamp. We sampled across three zones of one such swamp in the southern Peruvian to test the following hypotheses: 1) magnitudes of CH_4_ emission are greater in high productivity palm-dominated neotropical peatlands compared to lower productivity systems covered by herbaceous vegetation that provides less carbon substrate; and 2) tropical peatlands with low pH or high phenolics content will emit less CH_4_ than circum-neutral pH or low phenolic sites because of associated constraints on decomposition. We also summarize the few published data from neotropical peatlands to estimate the aggregate CH_4_ source strength of these ecosystems and assess sources of variability between them.

## Methods

### Study site

We collected field data for this study from a 250 ha peatland near the Los Amigos Biological Station, or Centro de Interpretacion y Capacitacion de Rio los Amigos (subsequently referred to as ‘CICRA peatland’) in mid-December 2016. Householder et al (2012) describe in detail the hydrogeomorphic setting of this and other peatlands in the Madre de Dios region. We briefly summarize the critical details here. The CICRA peatland abuts a steep terrace escarpment and is fed by numerous small perennial seeps. The regional climate has wet and dry seasons, but since the peatland is fed by groundwater, it is believed to remain inundated year round. The canopy is dominated by the palm *Mauritia flexuosa* which thrives in areas with permanently saturated soils throughout lowland neotropical forests from Panama to southern Brazil [14]. Formal floristic surveys of the CICRA peatland have yet to be published. A map of CICRA peatland bathymetry reveals the presence of three distinct components: two basins with peat depth exceeding 900 cm and ‘Intrabasin Flats’ with much shallower peat depth of 100 to 200 cm. In the Intrabasin Flats canopy coverage of *M*. *flexuosa* abruptly decreases from more than 85% to less than 10% giving way to open *Cyperaceae* mires. The extent to which emergent vegetation contributes to peat formation in these ecosystems is not clear, but the association between high palm density and deep peats is consistent with the conventional wisdom that the dense underground root system of *M*. *flexuosa* is a major contributor to soil carbon accumulation [14].

**Fig 1:**
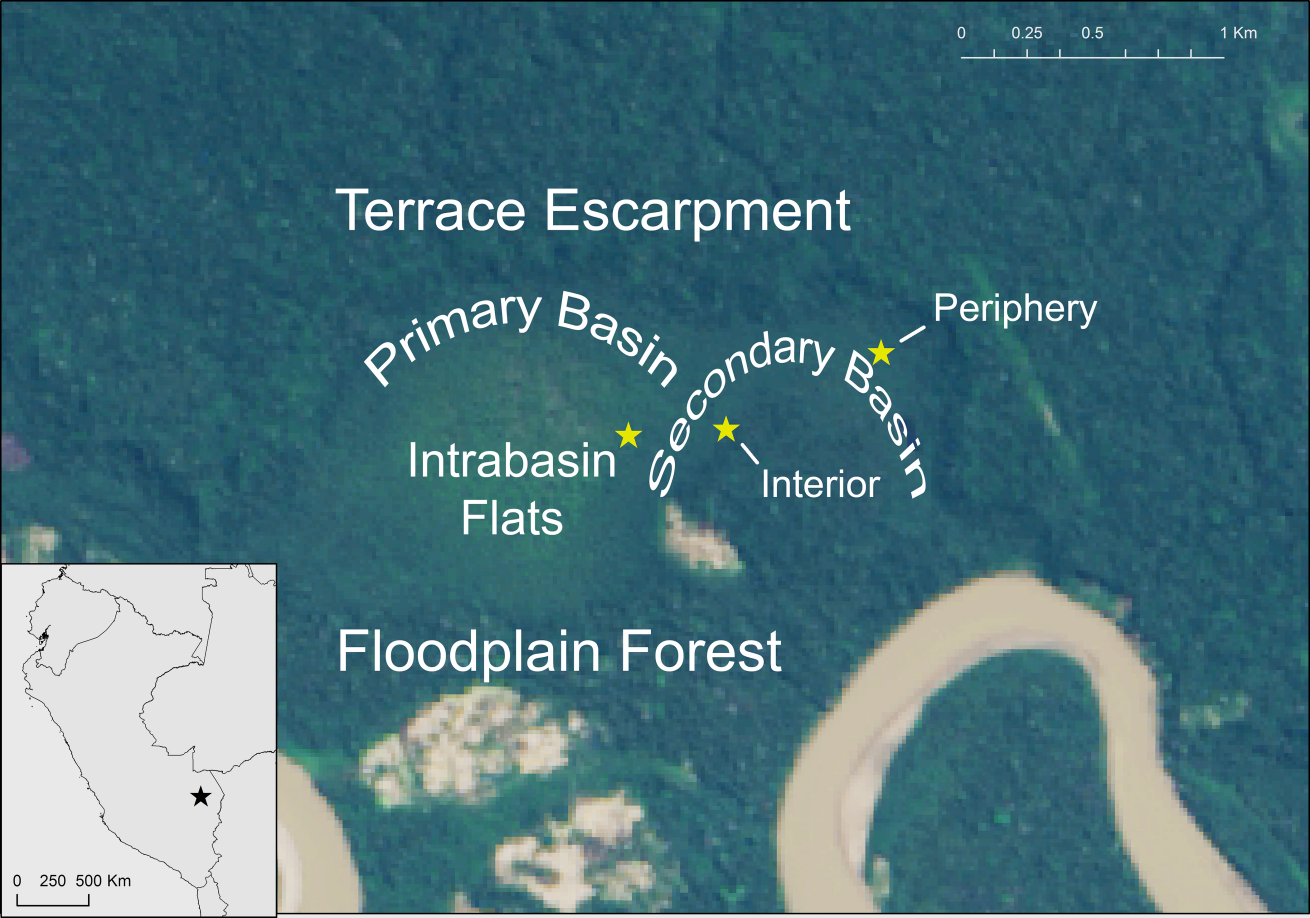
Map of the Los Amigos peatland in Madre de Dios, Peru, with sampling locations marked by stars. Basin Periphery: -12.55664 S, -70.1117 W; Basin Interior: -12.55926 S, -70.11702 W; Intrabasin Flats: -12.55947 S, -70.12037 W. Background image from ArcMap 10.3.

We divided our sampling effort between three zones of the CICRA peatland (Fig 1) in order to test hypotheses about the effects of vegetation type and minerotropic status. One sampling site was located in the secondary basin close to the terrace escarpment and groundwater seep sources (referred to as ‘Basin Periphery’). A second site was located in the secondary basin roughly 500 m from the terrace to represent a more ombrotrophic but otherwise similar system in terms of vegetation and peat depth (‘Basin Interior’). The third site was located in an open herbaceous zone of the Intrabasin Flats.

### Sample collection

#### Soils

To test for the presence of a minerotrophic-ombrotropic gradient from peatland periphery to interior, we measured a suite of soil chemical properties: total carbon, total nitrogen, total phosphorus, soluble phenolic compounds, extractable nitrate/nitrite, extractable ammonia/ammonium, and pH. From each site we extracted three replicate cores using a stainless steel peat box corer. We split each core into 5 cm sections in the field and stored them in sealed plastic bags for transport to the Duke University Wetland Center laboratory for analysis. We attempted to limit exposure of soil cores to oxygen, but any ammonium oxidation that may have occurred during sample transport would bias extractable nitrate/nitrite and extractable ammonia/ammonium values.

We measured total carbon and nitrogen content using an elemental analyzer (CE Instruments, Wigan, UK) and total phosphorus using a nitric-perchloric digestion and the molydenate blue spectrophotometric method [15]. We performed a 12-h deionized water extraction of soil subsamples and analyzed extracts for soluble phenolics following Lowe (1993) [16], and for nitrate/nitrite and ammonia/ammonium using a Lachat Quickchem 8000 autoanalyzer. We measured soil pH using both a 5:1 ratio of wet soils to DI water followed by the addition of 0.125 mL of CaCl_2_ following the methods outlined in Carter and Gregorich (2007) [17].

#### Methane

We used a static chamber method to measure CH_4_ flux [18,19]. We used 30 cm diameter plastic collars with water-fillable gutters and sampled using a rod for chamber top setup and sampling to avoid disturbing soil adjacent to chambers [20]. Rather than embed collars into the soil, as is often the practice, we simply rested them unanchored onto the soil surface in order to avoid unnecessary disturbance. This was possible because all sites were flooded with up to 10 cm of standing water. We placed six collars pseudo-randomly at each site [except four at the Secondary Basin periphery] avoiding areas where overlying vegetation or palm debris would interfere with chamber setup.

We extracted four 50 ml headspace samples from opaque chambers at 5–10 minute intervals for incubations lasting a total of 20 to 30 min and total extracted gas was roughly 1% of the 20 L headspace volume. We recorded internal chamber temperature at the time of each sample extraction and the height of each chamber in order to calculate gas concentration using the Ideal Gas Law. We stored samples in gas-tight mylar bags and transported them to the Duke University Wetland Center laboratory in North Carolina, USA for analysis within one week on a Varian 450 gas chromatograph. We calculated flux using linear regression of headspace concentration over time, which yielded r-squared values of at least 0.95 in all cases.

We measured the concentration of CH_4_ in soil pore water using a headspace equilibrium method [21]. We collected 4 replicates samples of 40 ml pore water from 10–15 cm soil depth at each site (except 2 replicates from Secondary Basin periphery) using a custom-made slotted metal sipper and injected them directly, without exposure to ambient air, into mylar bags pre-loaded with 100 ml of dinitrogen (N_2_). Dissolved gases were allowed to equilibrate with headspace N_2_ during the 48 to 72 hours of transport time to the Wetland Center laboratory, at which point 60 ml of headspace gas was extracted and deposited into an empty mylar bag for dry storage until samples could be analyzed on a Varian 450 gas chromatograph within one week. We used Henry’s Law to calculate the concentration of CH_4_ in pore water based on the concentration of CH_4_ measured in headspace.

### Statistical analyses

We tested for differences in mean CH_4_ flux between the Secondary Basin Periphery, Secondary Basin Interior and Intrabasin Flats using Analysis of Variance (ANOVA). Finding that mean CH_4_ flux was not equal across the three areas, we followed up with the pairwise post-hoc Tukey’s test of honest significant differences. We performed a Welch’s t-test to test for differences in mean CH_4_ flux between the Secondary Basins (Periphery and Interior data combined) and the Intrabasin Flats. Since we lacked sufficient replication to compare mean pore water dissolved CH_4_, we lump the two basins sites and compare means of Secondary Basin versus Intrabasin Flats via a Welch’s t-test. We compared peat soil variables among sites using a Tukey’s honest significant difference test. All statistics were calculated using the R programming language [22].

### Literature synthesis and extrapolation

We searched Google Scholar and ISI Web of Science for publications matching the terms “methane mauritia -mauritius” or “methane amazon” to find ground-based measurements of CH_4_ emissions from South American peatlands and/or palm swamps. We excluded several studies pertaining to floodplain mineral soil systems [23–28]. We included a study of a *Raphia taedigera* palm peat swamp in Panama [29], despite its location outside of continental South America and the Amazon basin, because of the climatic similarity and geographic proximity.

To estimate the total annual contribution of neotropical peatlands to annual CH_4_ emissions we multiply the mean and standard error of published emissions rates from our literature synthesis by the recent estimate of 750,000 km^2^ of peatlands in tropical/subtropical Central and South America [6].

## Results

**Fig 2:**
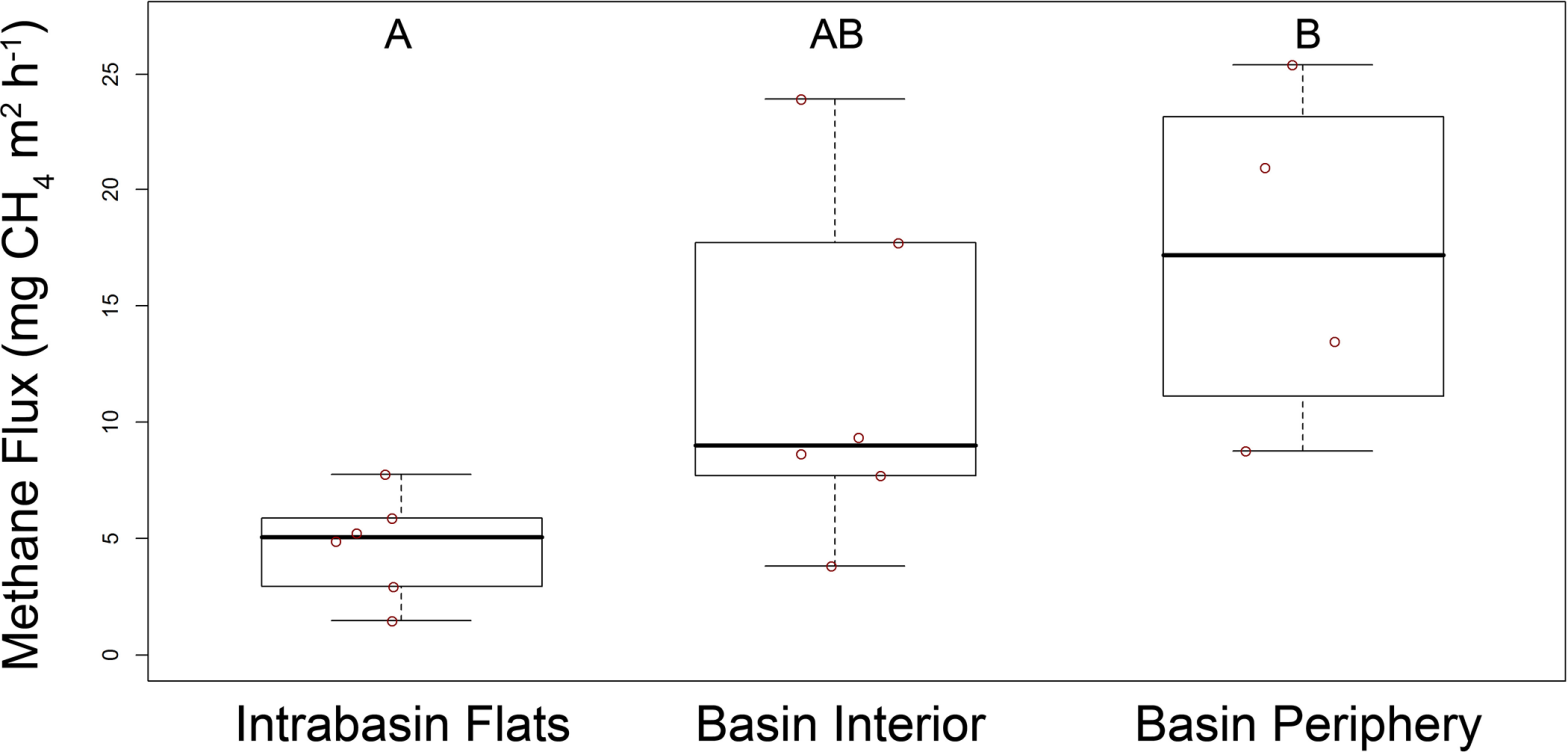
Tukey’s boxplots of methane emissions data from three sites at the Los Amigos peat swamp in Madre de Dios, Peru. Whiskers extend to data within 1.5 of intra-quartile range; no outliers were removed. Letters correspond to results of Tukey’s honest significant differences test at α = 0.05. The data used to generate this figure can be found in S1 Table.

We found that emissions were highly variable at the local scale within the CICRA peatland (ANOVA p = 0.019). Mean CH_4_ emissions at the Intrabasin Flats of 4.7 ± 0.9 mg CH_4_ m^2^ h^-1^ were significantly lower than those of 17.2 ± 3.7 mg CH_4_ m^2^ h^-1^ at the Secondary Basin Periphery according to Tukey’s honest significant differences test (p < 0.02) (Fig 2). Mean emissions at the Secondary Basin Interior of 11.9 ± 3.0 CH_4_ m^2^ h^-1^ were intermediate and not significantly different from emissions at the other sites (p = 0.13 and p = 0.39 for Intrabasin Flats and Secondary Basin Periphery, respectively). When we compared combined data from both basins to those of the Intrabasin Flats we found strong evidence for unequal mean CH_4_ emissions (Welch’s t-test; p = 0.004), with mean emissions from the basins roughly three times greater.

**Fig 3:**
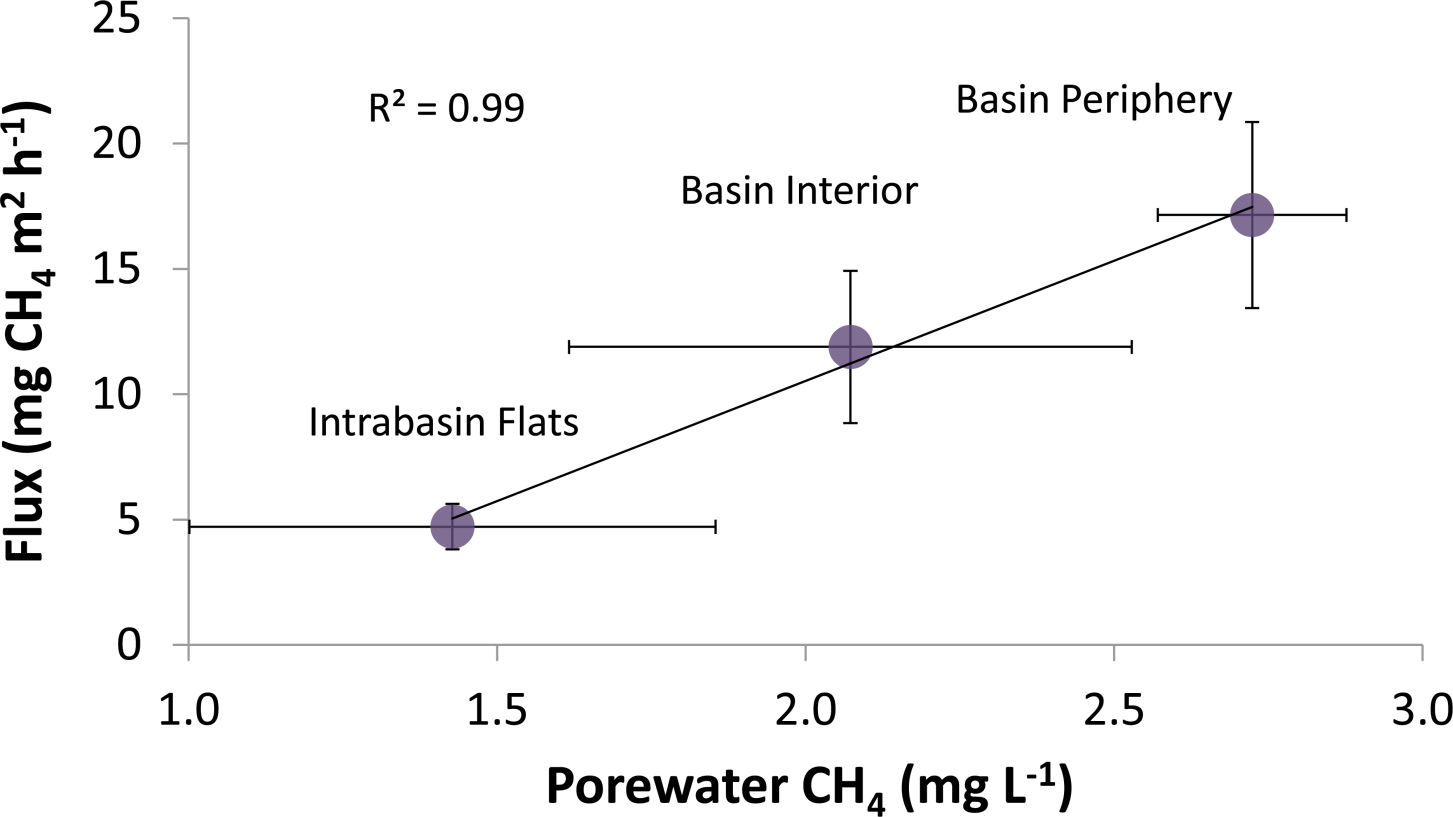
Mean (± standard error) methane emissions and methane dissolved in soil pore water from three sites at the Los Amigos peat swamp in Madre de Dios, Peru. The data used to generate this figure can be found in S1 Table.

Porewater CH_4_ concentrations followed a pattern mirroring that of emissions across the CICRA peatland. We found the two measures to be highly correlated (r^2^ = 0.99) after removing one outlier (for which porewater CH_4_ was more than double the next highest value from the data set) from the Secondary Basin Interior (Fig 3).

Many of the soil properties also showed significant variability between sites and may help explain site CH_4_ flux differences. The Intrabasin Flats stood out from one or both of the secondary basin sites for having significantly higher total phosphorus, soluble phenolics and pH (see Table 1). Soils of the two basin sites appeared to be more similar though the periphery had significantly less total nitrogen and carbon compared to the interior site.

**Table 1:** Mean (± standard deviation) surface soil (0–40 cm) chemistry (total nitrogen, total carbon, total phosphorus, phenolic compounds, extractable nitrate/nitrite, extractable ammonia/ammonium, water pH, salt pH) from three sites at the Los Amigos peat swamp in Madre de Dios, Peru. Letters (^a,b^) refer to Tukey groupings and bold indicates where Tukey’s honest significant differences were found between sites. The data used to generate these values can be found in S2 Table.

Site	TN	TC	TP	Phenol	NO_x_	NH_x_	pH aq	pH salt
	%	%	μg g^-1^	μg C g^-1^	μg g^-1^	μg g^-1^	-	-
Basin Periphery	**1.80**^**a**^	**33.9**^**a**^	832^a^	222^ab^	0.24^a^	0.57^a^	5.63^ab^	3.56^a^
	±0.20	±4.4	±74	±16	±0.02	±0.08	±0.10	±0.11
Basin Interior	2.18^b^	42.4^b^	801^a^	**208**^**a**^	0.21^a^	0.68^a^	**5.48**^**a**^	3.57^a^
	±0.19	±1.7	±155	±50	±0.17	±0.61	±0.22	±0.16
Interbasin Flats	2.31^b^	41.9^b^	**1060**^**b**^	**357**^**b**^	0.19^a^	0.56^a^	**5.92**^**b**^	**3.92**^**b**^
	±0.19	±1.0	±51	±156	±0.20	±0.60	±0.25	±0.06

Overall the *M*. *flexuosa*-dominated Secondary Basin of the CICRA peatland emitted on average 14.0 ± 2.4 mg CH_4_ m^-2^ h^-1^, and the open-canopy Cyperacea-dominated Intrabasin Flats emitted 4.7 ± 0.9 mg CH_4_ m^-2^ h^-1^. By averaging these values with those reported for other neotropical peatlands (Table 2), we estimate that 750,000 km^2^ of such systems [6] will emit 43 ± 11.9 Tg CH_4_ y^-1^. However, emissions rates vary by nearly an order of magnitude across sites despite the fact that almost all of data comes from Peru.

**Table 2:** Summary of published CH_4_ emissions data from neotropical peatlands.

Location	Peatland Type	CH4 flux	Source
		mg CH_4_ m^-2^ h^-1^	
Panama	*Raphia taedigera* Swamp	7.1	Wright et al 2013 [29]
Peru	*Mauritia flexuosa* Swamp	8.9	Van Haren and Cadillo-Quiroz 2015 [30]
N. Peru	*Mauritia flexuosa* Swamp	2.0	Murphy et al 2016 [31]
N. Peru	Mixed Palm Swamp	2.9	Murphy et al 2016 [31]
S. Peru	*Mauritia flexuosa* Swamp	14.0	this study
S. Peru	Cyperaceae Swamp	4.7	this study
**Mean**		**6.6**	
**Std. Dev.**		**4.4**	

## Discussion

### Vegetation

We found little evidence to support the hypothesis that the magnitudes of CH_4_ emission from palm-dominated and open herbaceous neotropical peatlands are similar. Methane emissions were lower from the open-canopy herbaceous zone of the Intrabasin Flats compared to emissions from the adjacent palm-dominated Secondary Basin, but given that these are the only CH_4_ measurements available for this poorly studied peatland type, it is impossible to know whether our result is generalizable to other regions of Amazonia. Typically, higher productivity wetlands emit more CH_4_ because of increased inputs of carbon substrate [32] and based on high rates of peat accumulation, *M*. *flexuosa* systems are thought to be highly productive [33]. Therefore it is likely that higher net primary productivity in the palm-dominated versus herbaceous zones of the CICRA peatland may be driving the differences in CH_4_ emissions we observed, but further research on the productivity and CH_4_ of these systems is needed.

Neotropical peatlands have yet to be systematically classified into dominant vegetation zones, but based on remote sensing of the Pastaza-Marañon foreland basin peatland complex in northern Peru, palm swamps dominate, covering 78% of total peatland areas [34].

Pole forest, for which no CH_4_ data is available, and Open-canopy herbaceous areas, for which we provide the first CH_4_ emissions measurements, each cover roughly equal parts of the remaining 22% [34].

The finding that significant quantities of CH_4_ are emitted through *M*. *flexuosa* palm trunks also suggests that CH_4_ emissions from palm-dominated peatlands based on soil flux chambers alone may be underestimating total efflux rates by 20% [30]. Emissions via woody vegetation has been shown to account for 62 to 87% of ecosystem CH_4_ emissions in a southeast Asian peatland [35] and further research in a temperate wetland found that unlike soil emissions, water table fluctuations had a minimal impact of tree stem CH_4_ flux [36]. It is possible that emissions via *M*. *flexuosa* trunks are proportionally more important to net CH_4_ flux during dry seasons when CH_4_ oxidation can be high if surface soils become exposed. More work is needed to investigate how much CH_4_ is emitted through plants in other Amazonian peatlands and whether the relative strength of tree flux varies with hydrology as soil flux does.

### Soil properties

Although we found CH_4_ emissions to diminish along a distance gradient from groundwater seep sources, the ombrotrophic conditions we expected to find in the peatland interior proved not to exist. We actually found soil pH to be higher at the Intrabasin Flats, the opposite of the pattern we had predicted. Furthermore we found the Intrabasin Flats soils to have higher total phosphorus and nitrogen content compared to the basin sites closer to groundwater sources indicating that the hydrologic inputs to the center of the peatland are not dominated by precipitation. Thus distance from groundwater source turns out to be a poor predictor of nutrient limitation and trophic state in this context. It is likely the much shallower depth of the peat at the Intrabasin location could have enhanced the transfer of nutrients from the mineral substrate to the upper peat layers. However, the slightly higher pH and total nitrogen and phosphorus at the Intrabasin Flats cannot explain the low CH_4_ emissions and porewater concentrations we observed. Importantly, we did find significantly higher phenolic content in the Intrabasin Flats peat soil, which has been shown to decrease GHG fluxes and decomposition rates in southern compared to northern peatlands [12]. Further research is needed to test the importance of phenolics in tropical peatlands and our original hypothesis regarding ombrotrophic versus minerotrophic CH_4_ patterns in the neotropics. Truly ombrotrophic tropical peatlands are known to occur in northern Peru with coverage by M. flexuosa or pole forest [34,37] and our original hypothesis could be retested at these sites.

### Hydrology

At the CIRCRA peatland, groundwater seeps from the adjacent terrace apparently keep the site perennially inundated [14] creating hydrologic conditions conducive to potent CH_4_ emissions year round. The strong correlation between soil porewater dissolved CH_4_ and emissions we found at the CICRA peatland is typical of wetlands with a months-long history of inundation and minimal CH_4_ oxidation capacity of soils [38]. The hydrologic setting at CICRA contrasts with that of a palm-dominated peatland in Panama where water levels and CH_4_ emissions rates fluctuate in concert with patterns of precipitation, resulting in relatively low mean CH_4_ emissions because of regular oxidation of surface soils [39]. High frequency CH_4_ measurements at CICRA and other Amazonian peatlands coupled with automated hydrologic monitoring will be needed to clarify the relationship between CH_4_ emissions and hydrology.

The high porewater CH_4_ concentrations we found at the CICRA peatland may point to a potentially important source of underestimation in net ecosystem flux. Groundwater supersaturated with CH_4_ will feed into headwater streams where outgassing will occur from surfaces waters. This phenomenon has been documented in the Brazilian Amazon where researchers found high concentrations of CH_4_ in stream surface water which they attributed to allochthonous sources in the watershed [40].

### Geographic distribution

The spatial clustering of CH_4_ studies in Peru (and the additional Panama site lying outside of the Amazon region) poses a serious regional bias problem in our analysis. The other data are sourced from northern Peru so our study in southern Peru does increase the spatial coverage, but CH_4_ dynamics of much of Amazonia’s peatlands remain unstudied. We also found in our data review that CH_4_ emissions rates from neotropical peatlands vary by nearly an order of magnitude. High variability combined with a small sample size of sites makes our estimate for the total contribution of these ecosystems to the global CH_4_ budget highly uncertain, but provides some of the first field evidence for the potential importance of these peatlands to the global CH_4_ budget. The latest study of the distributions of neotropical peatlands asserts that Colombia has an area of peat coverage comparable to that of Peru, while four times more exists in Brazil [6]. No studies of peatland CH_4_ emissions have been published to date in Brazil or Colombia, two of the world’s top four countries by tropical peatland area. Access is a major limitation as the vast majority of Amazonian peatland sites lie far from transportation infrastructure. Given the above noted constraints we estimate that the newly recognized vast areas of neotropical peatlands [6] may contribute 4.9 to 8.6 percent of the global CH_4_ budget of 645 Tg CH_4_ y^-1^ [41] based on the available ground-based data. This estimate is a first effort to bridge the ‘missing link’ of these tropical wetlands [2], but it will likely need significant revision as future studies lend additional data to the limited data set.

### Conclusions

Methane fluxes were variable across the peat soil chemical gradient found in the swamp peatlands at Madre de Dios, Peru. Overall the more productive *M*. *flexuosa*-dominated swamp sites on deep peat emitted CH_4_ at three times the rate found in the shallower peat open-canopy Cyperacea-dominated flats. The lower rates were found at sites with higher phenolics, N and P content as well as higher pH. Improvements to our understanding of the relationship between hydrology, vegetation community, productivity, soil chemistry and CH_4_ emissions from these ecosystems will hopefully allow us to better extrapolate from inevitably sparse spatial and temporal flux measurements leading to further refinements in estimates of tropical fluxes of CH_4_.

The past decade of growth in atmospheric concentrations of CH_4_ appears to be associated with a tropical biogenic source, which highlights the need for further study of biogeochemical functioning of tropical wetlands [42]. Looming threats to Amazonia make the need for further research in these peatlands especially urgent. A recent study has found that Amazonian forests may be less resilient to drought and fire than previously thought, with floodplains representing an ‘Achilles heel’ [43]. In additional to potential impacts posed by climate change, unregulated mining [14] and the expansion of commercial agriculture are serious anthropogenic threats to the hydrologic and ecologic integrity of Amazonian peatlands [44]. It will be critical to understand the ecological and biogeochemical functioning of these ecosystems before they are fundamentally altered by anthropogenic change.

## Supporting information

S1 Table Methane emissions and dissolved methane in porewater at 10 to 15 cm depth from three sites at the CICRA Peatland in Madre de Dios, Peru. (XLSX)

S2 Table Soil chemistry data from three sites at the CICRA in Madre de Dios, Peru. (XLSX)

## References

[1] Bousquet P, Ciais P, Miller JB, Dlugokencky EJ, Hauglustaine D a, Prigent C, et al Contribution of anthropogenic and natural sources to atmospheric methane variability. Nature. 2006 9 28;443(7110):439–43. doi: 10.1038/nature05132 17006511 10.1038/nature05132

[2] Sjögersten S, Black CR, Evers S, Hoyos-santillan J, Wright EL, Turner BL. Tropical wetlands: A missing link in the global carbon cycle? Global Biogeochem Cycles. 2014;28:1371–86. doi: 10.1002/2014GB004844 26074666 10.1002/2014GB004844 PMC4461074

[3] Bloom AA, Palmer PI, Fraser A, Reay DS, Frankenberg C. Large-Scale Controls of Methanogenesis Inferred from Methane and Gravity Spaceborne Data. Science. 2010 1;327:322–5. doi: 10.1126/science.1175176 20075250 10.1126/science.1175176

[4] Martinson GO, Werner F a., Scherber C, Conrad R, Corre MD, Flessa H, et al Methane emissions from tank bromeliads in neotropical forests. Nat Geosci. Nature Publishing Group; 2010;3(11):766–9.

[5] Carmichael MJ, Bernhardt ES, Bräuer SL, Smith WK. The role of vegetation in methane flux to the atmosphere: should vegetation be included as a distinct category in the global methane budget? Biogeochemistry. 2014 3 21;119(1–3):1–24.

[6] Gumbricht T, Roman-Cuesta RM, Verchot L, Herold M, Wittmann F, Householder E, et al An expert system model for mapping tropical wetlands and peatlands reveals South America as the largest contributor. Glob Chang Biol. 2017; 10.1111/gcb.13689 28295834

[7] Kayranli B, Scholz M, Mustafa A, Hedmark Å. Carbon Storage and Fluxes within Freshwater Wetlands: a Critical Review. Wetlands. 2009 12 9;30(1):111–24.

[8] Lawson IT, Kelly TJ, Aplin P, Boom A, Dargie G, Draper FCH, et al Improving estimates of tropical peatland area, carbon storage, and greenhouse gas fluxes. Wetl Ecol Manag. 2015;23(3):327–46.

[9] Whalen SC. Biogeochemistry of Methane Exchange between Natural Wetlands and the Atmosphere. Environ Eng Sci. 2005;22(1):73–93.

[10] Laanbroek HJ. Methane emission from natural wetlands: interplay between emergent macrophytes and soil microbial processes. A mini-review. Ann Bot. 2010 1;105(1):141–53. doi: 10.1093/aob/mcp201 19689973 10.1093/aob/mcp201 PMC2794055

[11] Freeman C, Ostle N, Kang H. An enzymic “latch” on a global carbon store. Nature. 2001 1 11;409(6817):149. 10.1038/35051650 11196627

[12] Wang H, Richardson CJ, Ho M. Dual controls on carbon loss during drought in peatlands Dual controls on carbon loss during drought in peatlands. Nat Clim Chang. 2015;5:584–7.

[13] Ye R, Jin Q, Bohannan B, Keller JK, McAllister S a., Bridgham SD. pH controls over anaerobic carbon mineralization, the efficiency of methane production, and methanogenic pathways in peatlands across an ombrotrophic–minerotrophic gradient. Soil Biol Biochem. Elsevier Ltd; 2012 11;54:36–47.

[14] Householder JE, Janovec JP, Tobler MW, Page S, Lähteenoja O. Peatlands of the madre de dios river of peru: Distribution, geomorphology, and habitat diversity. Wetlands. 2012;32(2):359–68.

[15] Wetzel RG, Likens GE. Limnological analyses. 3rd ed New York, NY, USA: Springer; 2000. 429 p.

[16] Lowe L. Water-soluble phenolic materials In: Carter MR, editor. Soil Sampling and Methods of Analysis. Boca Raton, Florida: Lewis Publishers; 1993.

[17] Carter MR, Gregorich EG. Soil Sampling and Methods of Analysis. 2nd ed Boca Raton, Florida: CRC Press; 2007.

[18] Livingston GP, Hutchinson GL. Enclosure-based measurement of trace gas exchange: applications and sources of error In: Matson A, Harriss RC, editors. Biogenic Trace Gases: Measuring Emissions from Soil and Water. Cambridge, MA, USA: Blackwell Science; 1995 p. 14–51.

[19] Weishampel P, Kolka R. Measurement of methane fluxes from terrestrial landscapes using static, non-steady state enclosures In: Hoover CM, editor. Field Measurements for Forest Carbon Monitoring. Springer Science & Business Media; 2008 p. 163–70.

[20] Winton RS, Richardson CJ. A cost-effective method for reducing soil disturbance-induced errors in static chamber measurement of wetland methane emissions Wetl Ecol Manag. Springer Netherlands; 2015 11 19;

[21] Kampbell DH, Wilson JT, Vandegrift S a. Dissolved Oxygen and Methane in Water by a GC Headspace Equilibration Technique. Int J Environ Anal Chem. 1989 8;36(4):249–57.

[22] R Core Team. R: A language and environment for statistical computing. R Foundation for Statistical Computing, Vienna, Austria; 2016.

[23] Belger L, Forsberg BR, Melack JM. Carbon dioxide and methane emissions from interfluvial wetlands in the upper Negro River basin, Brazil. Biogeochemistry. 2011;105(1):171–83.

[24] Wassmann R, Thein UG, Whiticar MJ, Rennenburg H, Seiler W, Junk WJ. Methane emissions from the Amazon Floodplain: Characterization of production and transport. Global Biogeochem Cycles. 1992;6(1):3–13.

[25] Bartlett KB, Crill PM, Sebacher DI, Harriss RC, Wilson JO, Melack JM. Methane Flux From the Central Amazonian Floodplain. J Geophys Res. 1988;93(D2):1571–82.

[26] Devol AH, Richey JE, Forsberg BR, Martinelli L a. Seasonal dynamics in methane emissions from the Amazon River floodplain to the troposphere. 1990;95.

[27] Devol AH, Richey JE, Clark WA, King SL, Martinelli LA. Methane emissions to the troposphere from the Amazon floodplain. Geophys Res. 1988;93:1583–92.

[28] Marani L, Alvalá PC. Methane emissions from lakes and floodplains in Pantanal, Brazil. Atmos Environ. 2007 3;41(8):1627–33.

[29] Wright EL, Black CR, Turner BL, Sjögersten S. Environmental controls of temporal and spatial variability in CO2 and CH4 fluxes in a neotropical peatland. Glob Chang Biol. 2013;19(12):3775–89. doi: 10.1111/gcb.12330 23873747 10.1111/gcb.12330

[30] Van Haren JLM, Cadillo-Quiroz H. Methane Flux of Amazonian Peatland Ecosystems: Large Ecosystem Fluxes with Substantial Contribution from Palm (maritia Flexuosa) STEM Emissions. In: American Geophysical Union 2015. p. #B43J-06.

[31] Murphy W, Berrio JC, Boom A, Page S, Arn Teh Y. Temporal variability in methane fluxes from tropical peatlands within the Peruvian Amazon. In: EGU General Assembly 2016. p. 16391.

[32] Whiting G, Chanton J. Primary production control of methane emission from wetlands. Nature. 1993;364:794–5.

[33] Lahteenoja O, Ruokolainen K, Schulman L, Oinonen M. Amazonian peatlands: an ignored C sink and potential source. Glob Chang Biol. 2009;15:2311–20.

[34] Draper FC, Roucoux KH, Lawson IT, A Mitchard ET, Honorio Coronado EN, Lähteenoja O, et al The distribution and amount of carbon in the largest peatland complex in Amazonia. Environ Res Lett. IOP Publishing; 2014;9:124017.

[35] Pangala SR, Moore S, Hornibrook ERC, Gauci V. Trees are major conduits for methane egress from tropical forested wetlands. New Phytol. 2013;197(2):524–31. doi: 10.1111/nph.12031 23253335 10.1111/nph.12031

[36] Pangala SR, Hornibrook ERC, Gowing DJ, Gauci V. The contribution of trees to ecosystem methane emissions in a temperate forested wetland. Glob Chang Biol. 2015;21(7):2642–54. 10.1111/gcb.12891 25665153

[37] Lähteenoja O, Ruokolainen K, Schulman L, Alvarez J. Amazonian floodplains harbour minerotrophic and ombrotrophic peatlands. Catena. Elsevier B.V.; 2009;79(2):140–5.

[38] Winton RS, Richardson CJ. Top-down control of methane emission and nitrogen cycling by waterfowl. Ecology. 2017;98(1):265–77. doi: 10.1002/ecy.1640 27918615 10.1002/ecy.1640

[39] Wright EL, Black CR, Cheesman AW, Drage T, Large D, Turner BL, et al Contribution of subsurface peat to CO2 and CH4 fluxes in a neotropical peatland. Glob Chang Biol. 2011;17(9):2867–81.

[40] Neu V, Neill C, Krusche A V. Gaseous and fluvial carbon export from an Amazon forest watershed. Biogeochemistry. 2011;105(1):133–47.

[41] Kirschke S, Bousquet P, Ciais P, Saunois M, Canadell JG, Dlugokencky EJ, et al Three decades of global methane sources and sinks. Nat Geosci. 2013;6(9):813–23.

[42] Nisbet EG, Dlugokencky EJ, Manning MR, Lowry D, Fisher RE, France JL, et al Rising atmospheric methane: 2007–2014 growth and isotopic shift. Global Biogeochem Cycles. 2016;30:1356–70.

[43] Flores BM, Holmgren M, Xu C, van Nes EH, Jakovac CC, Mesquita RCG, et al Floodplains as an Achilles’ heel of Amazonian forest resilience. Proc Natl Acad Sci. 2017;201617988. 10.1073/pnas.1617988114 PMC5410816 28396440

[44] Roucoux KH, Lawson IT, Baker TR, Del Castillo Torres D, Draper FC, Lähteenoja O, et al Threats to intact tropical peatlands and opportunities for their conservation. Conserv Biol. 2017; 10.1111/cobi.12925 PMC6849624 28272753

